# Effect of temperature on functional response of *Blattisocius mali* (Acari: Blattisociidae) preying on the acarid mite *Tyrophagus putrescentiae*

**DOI:** 10.1038/s41598-025-00268-z

**Published:** 2025-05-02

**Authors:** Katarzyna Michalska, Manoj Kumar Jena, Marcin Studnicki

**Affiliations:** 1 Department of Plant Protection, Institute of Horticultural Sciences, Warsaw University of Life Sciences, Nowoursynowska 159, Warsaw, 02-776 Poland; 2 Department of Biometry, Institute of Agriculture, Warsaw University of Life Sciences, Nowoursynowska 159, Warsaw, 02-776 Poland

**Keywords:** Acarid mite, Attack rate, *Blattisocius mali*, Handling time, Mesostigmata, Potential for prey mortality, Predation, Ecology, Zoology

## Abstract

**Climate warming significantly impacts soil temperature and moisture**,** leading to changes in the activity of soil mites and the foraging behaviour of edaphic predatory mites. The current research aimed to investigate the effect of temperature on the functional response of the predatory soil mite** ***Blattisocius mali*** **Oudemans preying on either eggs or males of the mould mite** ***Tyrophagus putrescentiae*** **Schrank. To analyze the functional response type**,** the generalized functional response equation of Real was used while the functional response parameters were determined using Roger**,** Hassell**,** and Cabello** ***et al.*** **models. Female adult** ***B. mali*** **displayed Type III and Type II functional responses when preying on eggs and males**,** respectively across all tested temperatures**,** ranging between 10 **°C** and 35 **°C**. The handling time of** ***B. mali*** **was shorter at higher temperatures**,** 25 **°C,** 30 **°C,** and 35 **°C** when preying on either eggs or males. In contrast**,** the potential for prey mortality**,** the attack rate**,** and the functional response ratio were higher at higher temperatures indicating higher efficiency of** ***B. mali*** **at higher temperatures. The temperature strongly impacted predators’ efficiency**,** as accelerated predator action under warming increased prey consumption. However**,** functional response type did not change with warmer temperatures but varied with changing prey stages from egg to male.**

Ongoing climate change is projected to raise global temperatures by 2 to 8 ^o^C over the next century, with atmospheric CO_2_concentrations expected to reach 800 ppm^1^. This climate warming is leading to changes in precipitation patterns, which can directly impact soil temperature and moisture levels^2^. Temperature can impact the biology, population dynamics^3,4^, abundance, species diversity, and richness of soil mite communities^5^. It also influences the metabolic rate, feeding, locomotor, and searching activity of soil mites^6,7^. It is closely linked to ecosystem functions such as trophic interactions through the consumption and metabolism of the predator and prey^6,8,9^. It can affect predatory soil mites’ functional response which is one of the important aspects of quantifying trophic interactions^10,11^.

The functional response is a critical component of the interaction between predator or parasitoid and prey or host, playing a key role in the dynamics of animal populations and ecological communities^11^^–^^18^. It illustrates how the number of prey captured by a predator or host parasitized by a parasitoid changes with the density of prey or host available in the environment. There are three basic types of functional response, Type I, II, and III, described by Holling^11^. Over the years, researchers have suggested various modifications to these basic types^16–18^. Type I shows a linear increase in consumption rate until it reaches a plateau; Type II demonstrates a hyperbolic approach to the maximum consumption rate as prey density rises; Type III involves an initial rise in consumption rate, followed by a decrease after reaching a turning point on a sigmoid curve. In insect and mite predators, Type II and III responses are most frequently reported^13,14,19^. Additionally, there is a Type IV functional response, the domed type, which indicates a decrease in predation efficiency at specific prey densities; this has also been noted in predatory mites^20,21^.

The effectiveness of a predator can be measured by looking at the functional response parameters, which include the predator’s attack rate, the handling time^14^, and the predator’s potential for prey mortality^22^. Predators with high attack rates and short handling times are expected to be the most effective for biological control^23–25^. Alternatively, if a predator is very efficient and causes significant prey mortality, it is likely to have a high potential for mortality to its prey. On the other hand, if a predator is not as efficient and does not cause much mortality, its potential for prey mortality will be lower^22^. The ecological impact of the predator can be assessed by the functional response ratio (FRR) which is the attack rate or potential of prey mortality divided by the handling time^25,26^. This parameter is especially useful when handling time and attack rate give the opposite predictions. The higher the value of FRR, the higher the impact of the predator on the ecosystem and vice versa^26^. In invertebrate predators, the type and parameters of functional response can vary depending on host plant^27^, temperature^28,29^, humidity^25,30^, age of predator^31^, type of predator and prey^14,32,33^, and exposure to insecticides^34^.

Previous studies show that temperature has the potential to alter the type of functional response in insect and mite predators. For instance, rising temperature shifted the type of functional response from Type II to Type III in the pentatomid bugs *Podisus maculiventris* Say and *P. nigrispinus* Dallas (Hemiptera: Pentatomidae) preying on larvae of the beet armyworm *Spodoptera exigua *Hübner (Lepidoptera: Noctuidae)^29^. On the contrary, the rising temperature changed the functional response type from Type III to Type II in the phytoseiid mite *Amblyseius swirskii* Athias-Henriot (Acari: Phytoseiidae) foraging on eggs of the two-spotted spider mite *Tetranychus urticae *Koch (Acari: Tetranychidae)^28^ and the macrochelid mite *Macrocheles muscaedomesticae* Scopoli (Acari: Macrochelidae) feeding on eggs of the house fly *Musca domestica* L. (Diptera: Muscidae)^10^. On the other hand, the temperature change did not affect the type of functional response in the phytoseiid mite *Neoseiulus californicus* McGregor (Acari: Phytoseiidae) feeding on eggs, larvae, nymphs, or adults of *T. urticae*^35^. This suggests that temperature has varying impacts on the predator-prey system, probably due to species-specific differences in the sensitivity of predator and prey to temperature and foraging behaviour^36,37^. As temperature may destabilize interactions between predator and prey by either increasing predator activity or boosting prey mortality rates^38^, valuing the effect of temperature on species-specific responses of the predator-prey system can enable a better understanding of the impact of temperature on food webs.

Predatory mites belong to the family Blattisociidae (Acari: Mesostigmata) which inhabit a diverse array of habitats, including soil, mosses, grasses, and dead organic matter. They can also be found in association with fungi and various plant structures such as flowers, leaves, and tree bark, as well as within rodent and bird nests^4,39^. These mites are frequently linked to insects facilitating their movement to fragmented habitats^39–41^. Among the Blattisociid mites, the genus *Blattisocius*, including species such as *Blattisocius dentriticus* Berlese, *B. tarsalis* Berlese, *B. everti* Britto, Lopes and Moraes, *B. keegani* Fox, and *B. mali* Oudemans, is particularly well-studied. Although they commonly inhabit edaphic environments, outside of soil, litter, or rotten plant material, they are often reported in storage facilities, where they prey on coleopteran and lepidopteran pests as well as acarid mite pests associated with stored products^39^.

*Blattisocius dentriticus*, *B tarsalis*, *B. everti*, and *B. keegani *have been reported to have the potential to control mould mites^42–48^. Additionally, *B. mali* has been reported as a potential biocontrol agent of insects, nematodes, and mites^49–53^. Notably, the life table parameters of *B. mali* were much higher than those of *B. dentriticus*,* B. keegani*, or *Gaeolaelaps aculeifer* Raumilben (Acari: Laelapidae) while feeding on the mould mite *Tyrophagus putrescentiae* Schrank (Acari: Acaridae), which makes this predator an especially promising biological control agent against *T. putrescentiae*^54^. The acarid mites can cause serious problems in stored products, mushroom farms, and horticultural crops^55–58^. The *T. putrescentiae*is an omnivorous acarid mite and common in-house dust, soil with rotting plant material, and vertebrate nests. It is a pest of various stored food products and crop plants such as cucumber, gerbera, or bulbs of many ornamental plants^56,59^. It can develop in a wide range of temperatures, from 10 °C to 34 °C, and at an optimal temperature of 22 °C and humidity of 85%, it can make one generation in only 4.41 days^60,61^.

This study aimed to examine the effect of varying temperature levels on the functional response of *B. mali* preying on *T. putrescentiae*. This might not only enrich our knowledge about the possible negative effects of extreme temperatures on the stabilization of systems between soil predatory mites and acarid prey but also show at what temperatures this predator is most effective in the biological control of acarid mite pests. In the previous paper^25^, we demonstrated that a decrease in humidity level not only led to the decrease in *B. mali* predation rate on the *T. putrescentiae *eggs but also shifted its functional response from Type III to Type II on this prey. As the temperature has a considerable impact on blattisociid mite activity and development^4,62^, we hypothesized that this factor, similar to humidity, might significantly affect the interactions between *B. mali* and its prey, and the functional response of this predator. In our current research, we tested *B. mali* over a wide range of six temperature levels including extremes at 10 °C and 35 °C where this mite could still develop^63^. We also used two prey stages, eggs or adult males of *T. putrescentiae* to examine whether, and to which extent, the functional response of *B. mali* might change in the presence of smaller immobile eggs as prey or much bigger and movable males as prey, at varying temperature levels. Furthermore, we have compared different models based on the fitness of our data to provide a better understanding and interpretation of the dynamics within the predator-prey system.

## Results

**Fig. 1:**
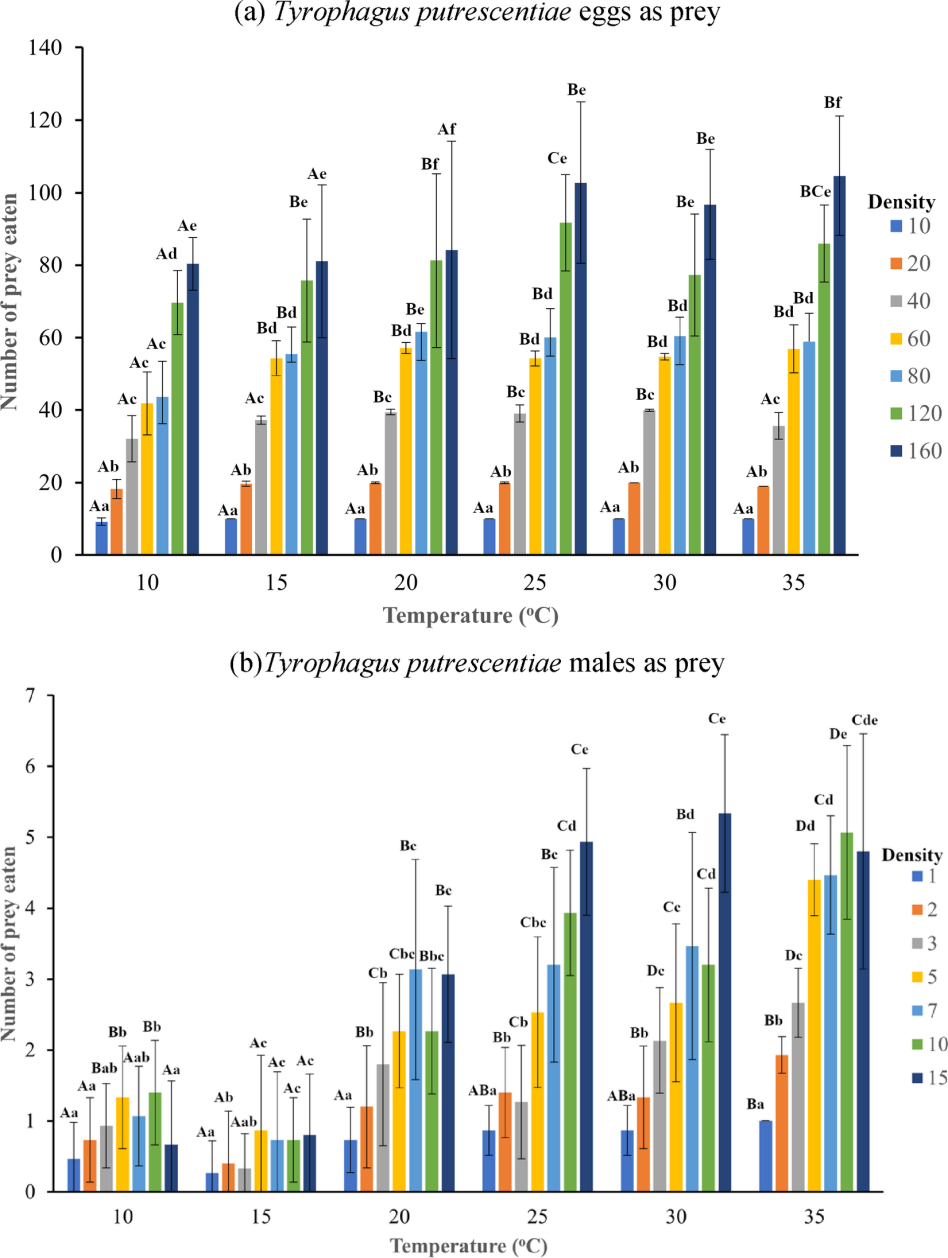
The effect of six temperatures and seven densities of *Tyrophagus putrescentiae* eggs or males on the mean number (±95% CI) of the *T. putrescentiae* eggs (**a**) or males (**b**) eaten by *Blattisocius mali *over 24 h period*. *Different lowercase or uppercase letters indicate significant differences between means (*P *< 0.05; Tukey test) for various prey densities within each temperature or among different temperatures, respectively.

The statistical analysis indicated a significant effect of both temperature (χ^2^ = 148.16; *df* = 5; *P* < 0.0001) and the density of *T. putrescentiae* eggs (χ^2^ = 925.60; *df* = 6; *P* < 0.0001) offered on the mean number of eggs eaten by *B. mali*. Moreover, there was a significant influence of both temperature (χ^2^ = 164.18; *df* = 5; *P* < 0.0001) and the density of *T. putrescentiae* males (χ^2^ = 103.45; *df* = 6; *P* < 0.0001) offered on the mean number of males eaten by *B. mali*. Furthermore, the interaction between temperature and density of prey eaten was found to be significant for both eggs (χ^2^ = 19.01; *df* = 30; *P* < 0.0001) and males (χ2 = 10.34; *df* = 30; *P* < 0.0001) as prey, indicating that the mean number of preys eaten by the predator depended not only on the temperature but also on the density of the prey offered. When *T. putrescentiae* eggs were offered as prey, the mean number of prey eaten by *B. mali* increased significantly with rising temperature across all tested prey densities except for 10 or 20 eggs (Fig. 1a). On the contrary, the mean number of *T. putrescentiae* males eaten by *B. mali* significantly decreased from 10 ^o^C to 15 ^o^C and then rose to 35 ^o^C in most prey densities (Fig. 1b).

The estimates of the parameters of the Real^64^ model showed that the value of the scaling component ‘q’ and handling time ‘T_h_’ were greater than zero for *T. putrescentiae* eggs as prey, indicating a Type III functional response at all tested temperatures (Table 1). On the other hand, the value of ‘q’ for *T. putrescentiae* males as prey was not significantly different from zero, and ‘T_h_’ was greater than zero across all tested temperatures, indicating a Type II response (Table 2).

**Table 1:** Estimates of various parameters of the Real^64^ model, a (attack rate), q (scaling component), and T_h_ (handling time), for the proportion of *Tyrophagus putrescentiae* eggs eaten by *Blattisocius mali* relative to the initial number of eggs provided at six temperatures over a 24 h period.

Temperature (^o^C)	Parameter	Estimate	Standard error	*P* value
10	a	8.8269	2.50950	0.0004
	q	0.5868	0.11521	< 0.0001
	T_h_	0.0014	0.00545	0.0184
15	a	1.2274	0.47540	0.0098
	q	0.6203	0.11532	< 0.0001
	T_h_	0.0164	0.00023	< 0.0001
20	a	0.8147	0.24739	0.0009
	q	0.7000	0.08172	< 0.0001
	T_h_	0.0112	0.00013	< 0.0001
25	a	8.8764	0.00035	< 0.0001
	q	0.0832	0.01181	< 0.0001
	T_h_	0.0081	0.00019	< 0.0001
30	a	4.5102	0.00076	< 0.0001
	q	0.2287	0.01391	< 0.0001
	T_h_	0.0088	0.00012	< 0.0001
35	a	4.7585	0.00076	< 0.0001
	q	0.0748	0.01391	< 0.0001
	T_h_	0.0081	0.00012	< 0.0001

**Table 2:** Estimates of various parameters of the Real^64^ model, a (attack rate), q (scaling component), and T_h_ (handling time), for the proportion of *Tyrophagus putrescentiae* males eaten by *Blattisocius mali* relative to the initial number of males provided at six temperatures over a 24 h period.

Temperature (^o^C)	Parameter	Estimate	Standard error	*P* value
10	a	1.9644	0.54479	0.0166
	q	0.0921	1.07088	0.3895
	T_h_	0.8846	0.12114	< 0.0001
15	a	1.3645	0.20633	0.0172
	q	0.0768	0.89782	0.9317
	T_h_	0.3799	0.51032	0.0149
20	a	1.4930	0.49234	0.0024
	q	0.2731	0.48173	0.5706
	T_h_	0.3136	0.05175	< 0.0001
25	a	1.5172	0.29906	< 0.0001
	q	0.4295	0.37430	0.2511
	T_h_	0.3208	0.16791	0.0069
30	a	1.8191	0.36637	0.0023
	q	0.2191	1.01921	0.4535
	T_h_	0.2417	0.38721	0.0018
35	a	4.4565	1.78402	0.0124
	q	0.2414	0.43564	0.5794
	T_h_	0.1844	0.01708	< 0.0001

**Fig. 2:**
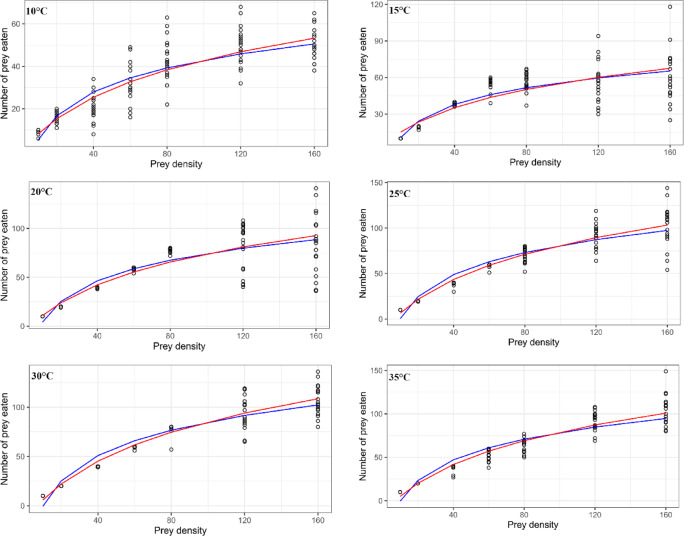
Type III functional responses of *Blattisocius mali* to the *Tyrophagus putrescentiae *eggs at six temperatures and seven prey densities predicted from the models. Blue and red lines were drawn based on the models proposed by Hassell^14^ and Cabello *et al.*^22^, respectively.

**Fig. 3:**
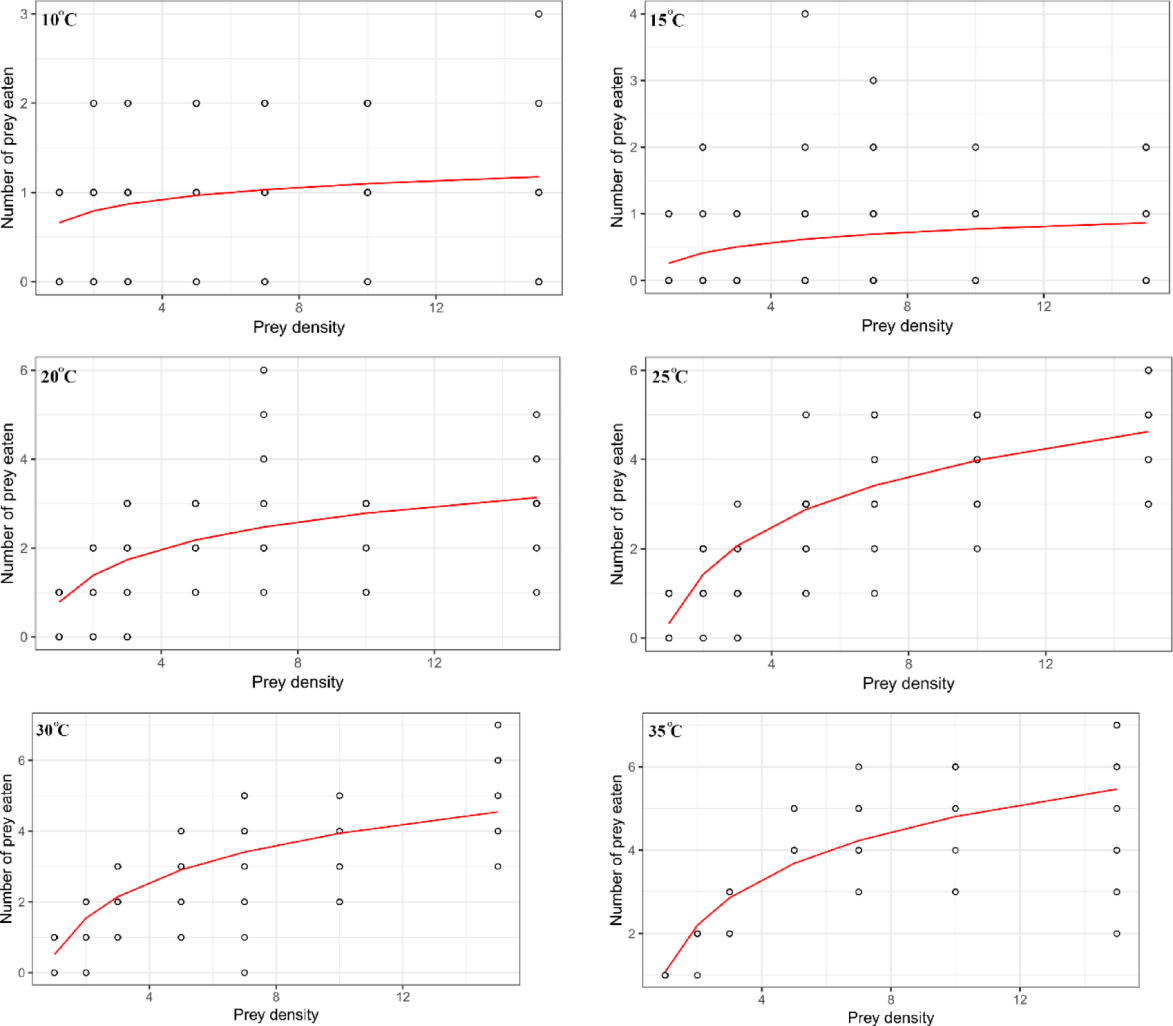
Type II functional responses of *Blattisocius mali* to the *Tyrophagus putrescentiae* males at six temperatures and seven prey densities predicted from the model proposed by Roger^65^.

The functional response curves were drawn and compared based on the models proposed by Hassell^14^ and Cabello et al.^22^ across all tested temperatures when *T. putrescentiae* eggs were used as prey. The comparison revealed parallel outcomes, indicating that the number of eggs eaten increased with increasing egg densities following a nearly sigmoidal shape (Fig. 2). On the other hand, when *T. putrescentiae* males were the prey, the curves were drawn based on the Roger^65^ model for all tested temperatures, indicating that the number of males eaten increased with increasing male densities following a hyperbolic fashion (Fig. 3).

**Fig. 4:**
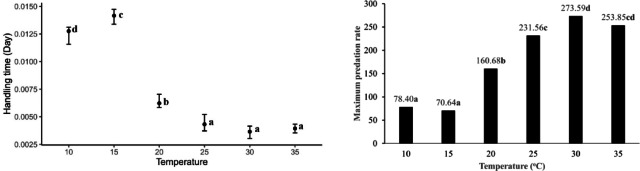
The handling time (T_h_) and maximum predation rate (T/T_h_) of *Blattisocius mali* preying on *Tyrophagus putrescentiae* eggs at six temperatures and seven egg densities, resulting from the Hassell^14^ model of Type III functional response. Different letters near the bars indicate significant differences between temperatures (*P* < 0.0001) based on 95% CI.

**Fig. 5:**
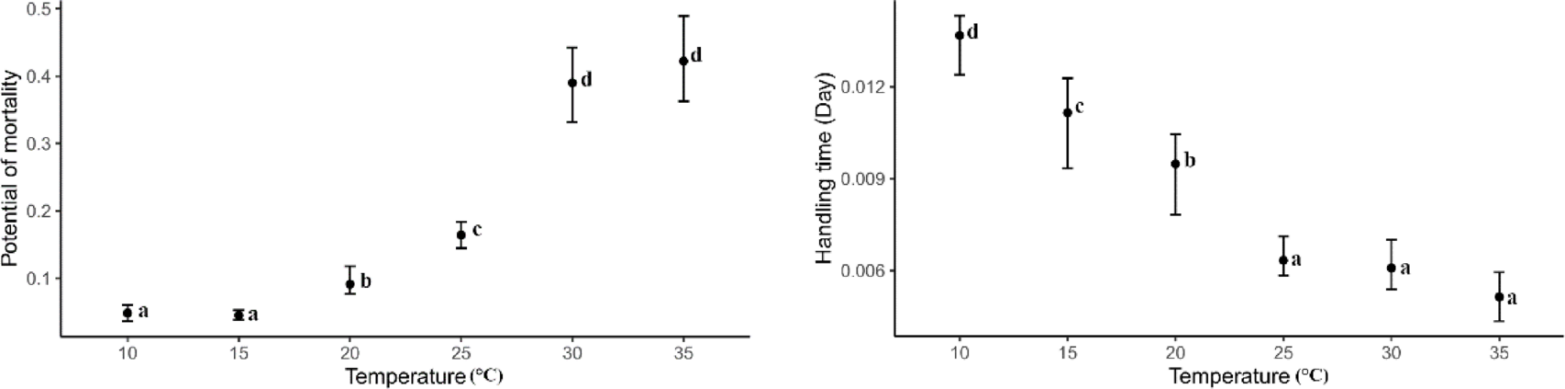
The potential of mortality (α) and handling time (T_h_) of *Blattisocius mali* preying on *Tyrophagus putrescentiae* eggs at six temperatures and seven egg densities, resulting from Cabello *et al.*^22^ model for Type III functional response. Different letters near the bars indicate significant differences between temperatures (*P *< 0.05) based on 95% CI.

Based on the Hassell^14^ model of Type III functional response, *B. mali* exhibited longer handling times at lower temperatures compared to higher temperatures when preying on *T. putrescentiae* eggs. However, at 10 °C, the handling time was significantly shorter compared to 15 °C while there were no significant differences in handling times at 25 °C, 30 °C, and 35 °C (Fig. 4). The maximum predation rate of *B. mali* was significantly influenced by warmer temperatures (χ^2^ = 43.16; *df* = 5; *P* < 0.0001). As the temperature rose, the maximum predation rate increased, peaking at 30 °C and 35 °C, where they remained statistically similar (*P* > 0.05) (Fig. 4). The parameters estimated from the Cabello et al.^22^ model showed that *B. mali* exhibited higher potential for prey mortality values and shorter handling times at higher temperatures when preying on *T. putrescentiae* eggs as compared to lower temperatures (Fig. 5). The values of potential for prey mortality were low and did not differ significantly at 10 °C and 15 °C (*P* > 0.05) while these values peaked but still did not show significant difference at 30 °C and 35 °C (*P* > 0.05). By contrast, the handling time was the longest at 10 °C, decreased with increasing temperature up to 25 °C, and then stabilized without further change up to 35 °C (*P* > 0.05) (Fig. 5).

**Fig. 6:**
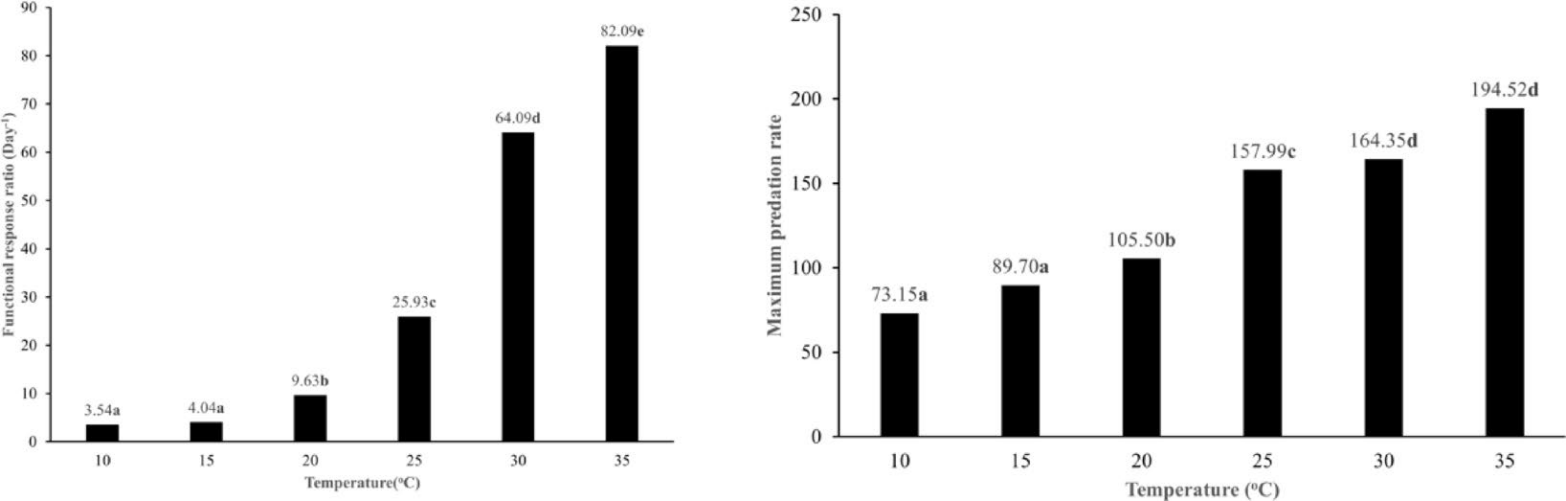
The functional response ratio (α/T_h_) and the maximum predation rate (T/T_h_) of *Blattisocius mali* preying on *Tyrophagus putrescentiae* eggs at six temperatures and seven egg densities, resulting from Cabello *et al.*^22^ model for Type III functional response. Different letters above the columns indicate significant differences between temperatures (*P* < 0.0001) based on the Dunn test with Bonferroni corrections.

Warming had a significant effect on both the FRRs (χ^2^ = 51.91; *df* = 5; *P* < 0.0001) and the maximum predation rates (χ^2^ = 33.28; *df* = 5; *P* < 0.0001) of the predator when exposed to *T. putrescentiae* eggs. The FRR was the lowest at 10 °C which did not vary significantly from that at 15 °C. However, these values were significantly increased at higher temperatures and achieved the highest at 35 °C (Fig. 6). Also, the maximum predation rates were lower at 10 °C and 15 °C, showing no significant difference (*P* > 0.05). In contrast, the maximum predation rates were higher at 30 °C and 35 °C, which also did not differ significantly from each other (*P* > 0.05) (Fig. 6).

**Fig. 7:**
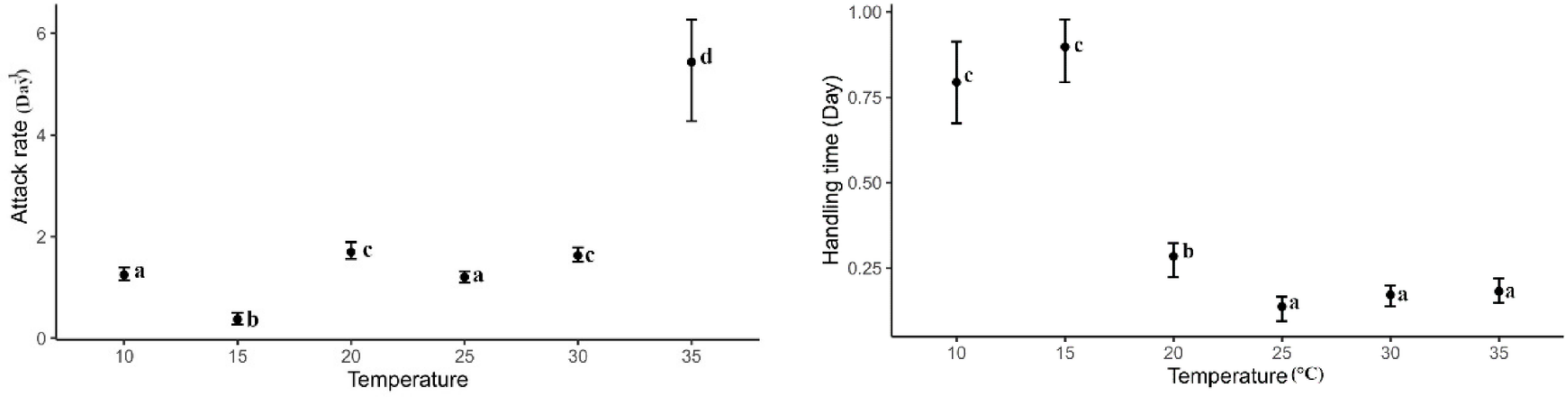
The attack rate and handling time of *Blattisocius mali* preying on*Tyrophagus putrescentiae* males at six temperatures and seven male densities, resulting from the Roger^65^ model of Type II functional response. Different letters near the bars indicate significant differences between temperatures (*P* < 0.05) based on 95% CI.

Based on the Roger^65^ model of Type II functional response, the attack rate of *B. mali* was significantly higher at 35 °C compared to other temperatures (*P* < 0.05) when preying on *T. putrescentiae* males. The attack rates fluctuated between 10 °C and 35 °C; it was significantly lower at 15 °C than at 10 °C. However, it increased again at 20 °C, slightly but significantly decreased at 25 °C, and then increased once more at 30 °C (*P* < 0.05) (Fig. 7). The handling times were longer at 10 °C and 15 °C, with no significant difference between them. It decreased from 15 °C to 25 °C and then remained consistent up to 35 °C (*P* > 0.05) (Fig. 7).

**Fig. 8:**
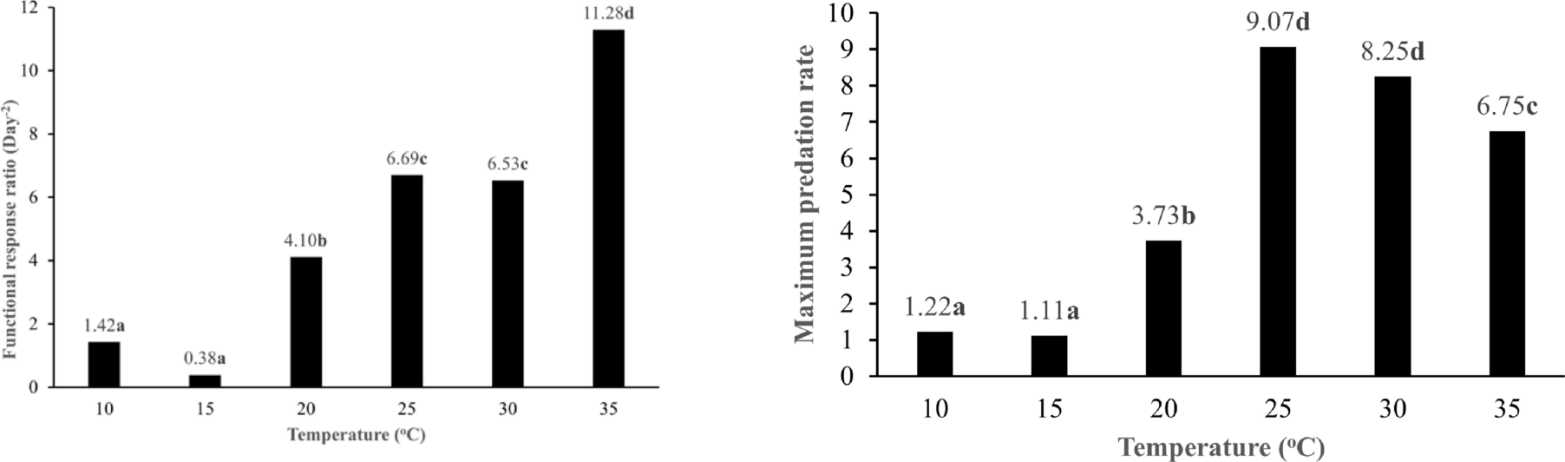
The functional response ratio (a/T_h_) and the maximum predation rate (T/T_h_) of *Blattisocius mali* preying on *Tyrophagus putrescentiae* males at six temperatures and seven male densities, resulting from Roger^65^ model of Type II functional response. Different letters above the columns indicate significant differences between temperatures (*P* < 0.0001) based on the Dunn test with Bonferroni corrections.

Temperature significantly affected both the FRRs (χ^2^ = 51.91; *df* = 5; *P* < 0.0001) and the maximum predation rates of *B. mali* when preying on *T. putrescentiae* males (χ^2^ = 33.28; *df* = 5; *P* < 0.0001) (Fig. 8). The FRRs were lower at 10 °C and 15 °C, with no significant difference between them (*P* > 0.05). However, the FRR showed an upward trend at elevated temperatures, reaching its peak at 35 °C. Similarly, the maximum predation rates were lower at 10 °C and 15 °C, where these values did not differ significantly (*P* > 0.05). On the other hand, as temperatures rose, the maximum predation rate increased, peaking at 25 °C; however, it declined at both 30 °C and 35 °C, with no significant variation between them (*P* > 0.05) (Fig. 8).

## Discussion

The present study demonstrated that the functional response of *B. mali* did not change with changing thermal conditions ranging between 10 °C and 35 °C but varied with changing prey stages, from egg to adult male of *T. putrescentiae*. The temperature ranges we tested are relevant across temperate or sub-tropical regions. Across all tested temperatures and prey densities, the predatory females exhibited Type III functional responses when *T. putrescentiae* eggs were used as prey and Type II responses when *T. putrescentiae* males were the prey. In addition, the handling times were shorter at 25 °C, 30 °C, and 35 °C compared to lower temperatures, regardless of whether the prey was either eggs or males. The potential for prey mortality and the maximum predation rate, estimated for eggs as prey, were the lowest at 10 °C and 15 °C but peaked at 30 °C and 35 °C. By contrast, the attack rate of the predator exposed to *T. putrescentiae* males showed fluctuation from 10 °C to 25 °C, with the highest rate occurring at 35 °C. The maximum predation rate was the lowest at 10 °C and 15 °C, peaked at 25 °C, then slightly decreased at 30 °C and 35 °C. For both prey stages, the FRRs increased with rising temperatures, recorded as the lowest at 10 °C and 15 °C and the highest at 35 °C.

In predatory insects and mites, the developmental stage of prey can influence the type of functional response^33,35,66^. Such a phenomenon has been also observed in this study. The females of *B. mali* exhibited Type III functional response when preying on *T. putrescentiae* eggs while Type II response when *T. putrescentiae *males were offered as prey. Also, when tested across varying humidity levels^25^, *B. mali* females initially followed a Type III response when preying on *T. putrescentiae* eggs; however, when the humidity dropped to a critical level of 33%, they transitioned to a Type II response. This raises an important question about the underlying mechanisms driving shifts in functional response types. In Type III functional response, the proportion of prey eaten initially increases, and generally, this type of functional response is expected when resources or environmental conditions are suboptimal^18,67,68^. As suggested by Hassell^14^, at low prey densities, there may be insufficient ‘reward rate” for a predator to continue the constant prey-searching activity. Factors like the necessity of learning to capture prey, the small size of the prey, effective defence mechanisms, or the availability of inaccessible refuges can all hinder predation efforts^18,67–70^. According to a study on life table parameters^54^, the eggs of the *T. putrescentiae* were less profitable prey for *B. mali* than larvae. It suggests that, unlike other prey stages, the eggs of *T. putrescentiae* may be a suboptimal prey stage for *B. mali* females, leading to a Type III functional response. The eggs are too small to satisfy hunger immediately, and are immobile, making them difficult for predators to detect, especially at low densities. However, the situation may change under worsening environmental conditions such as a drop in humidity. Low humidity may lead to substantial water loss in mites, including predatory soil mites. At 33% humidity, *B. mali* females significantly decreased predation rate, most presumably to conserve energy^25^. Nonetheless, they also shifted to Type II functional response, indicating that their efforts in searching for prey remained low regardless of whether the egg densities were high or low.

Similar to humidity, temperature also affects the functional response of insect and mite predators^10,28,29^. At suboptimal temperatures, the cost associated with searching for food may exceed foraging rewards due to longer handling times. Additionally, rising temperatures might lead to the shift from Type II to Type III functional response or vice versa^18,71,72^. Contrary to our initial hypotheses, *B. mali* females did not change the functional responses when preying on either *T. putrescentiae* eggs or males across the tested temperatures, including extremes of 10 °C and 35 °C. However, this does not mean that a shift in functional response will not occur in this predator if only the range of tested temperatures is widened even further from the optimal values. It should be emphasized that *B. mali* was tested under conditions of optimal humidity of 85 ± 5%. In another study involving a soil mite, *M. muscadomesticae* that was preying on eggs of *M. domesticae*, the shift from Type III to Type II functional response was noted at 33 °C^10^. However, the mite was deprived of food before the experiment and tested at a much lower humidity level of 65.5%. This combination of high temperature and low humidity might have affected both the searching rate and functional response of this predator.

Studying functional responses not only enhances our understanding of how predator-prey interactions can fluctuate at the population level but also sheds light on the factors that may disrupt the stability of these systems^18,67,71–73^. Type II and Type III functional responses show distinct differences in terms of the stability of the predator-prey system. Type II response is characterized by a gradual decrease in the proportion of prey killed, indicating inverse density dependence. By contrast, Type III responses exhibit positive density dependence up to a certain threshold prey density, which may help in stabilizing the system when the average prey densities fall below this threshold^18,67,74^. A recent study by Daugaard et al.^72^ on the effect of warming on the functional response of the ciliate predator, *Spathidium* sp. and its prey *Dexiostoma campylum* (Stokes) Jankowski (Hymenostomatida: Tetrahymenidae), have confirmed that shifts from Type III to Type II responses may destabilize the predator-prey system. Simulation studies on population dynamics indicated that shifting to a Type II response resulted in increased prey consumption at low densities, ultimately leading to extinction in nearly all scenarios. Our findings suggest that *T. putrescentiae *eggs which constitute nearly 50% of a prey population^75^, may play an important role in stabilizing the *B. mali*-acarid mite system. However, to verify this hypothesis, younger and smaller developmental stages of prey such as acarid mite eggs should be used in the tests on the functional response of *B. mali*.

The phenomenon of warming has been shown to accelerate the metabolic rate, feeding rate, and energy gain requirements^6,76^, which the predators may meet by consuming more prey, possibly explaining our results of increased predation observed under warming. *Tyrophagus putrescentiae *performed well within a wide range of temperatures from 20 °C to 32.5 °C^77^, promoting prey population growth and increasing prey availability which coincides with the higher predation by the predator. The increased predation under warming has been observed for the predatory mites *M. muscaedomesticae* preying on the immatures of *M. domestica*^78^; *A. swirskii* preying on eggs of *T. urticae*^28^; *Neoseiulus barkeri* Hughes (Acari: Phytoseiidae) preying on nymphal stages of *T. urticae*^79^; *Amblyseius longispinosus* Evans (Acari: Phytoseiidae) preying on active life stages of the bamboo spider mite *Aponychus corpuzae *Rimando (Acari: Tetranychidae)^80^, indicating the widespread nature of this phenomenon.

The magnitude of functional response can be described by the predator’s attack rate, handling time, and maximum predation rate^14^. In this study, we also used the potential of prey mortality (α), a parameter of the expression for the Hassell^14^ Type III functional response model developed by Cabello et al.^22^. In alignment with our previous study^25^, this model fitted well with our data on the functional response of *B. mali* when preying on *T. putrescentiae* eggs. Also, α, which corresponds to the potential of prey mortality in a Type III response turned out to be a useful parameter in the interpretation of the effectiveness of *B. mali* exhibiting Type III functional response. In our study, handling time was lower while the attack rate and potential of mortality was higher at higher temperatures which might be associated with the higher moving activity, metabolic rate, energy demands, and food intake by the predator *B. mali*^6,7^. Interestingly, the effectiveness of the predator varied significantly at lower temperatures, specifically between 10 °C and 20 °C, depending on the stage of prey. For eggs as prey, the potential for prey mortality increased steadily with rising temperatures. In contrast, when preying on *T. putrescentiae *males, the instantaneous attack rate initially showed a slight increase before declining, exhibiting fluctuations until 25 °C, after which a marked increase was observed as temperatures rose to 35 °C. The fluctuations in attack rate might be associated with the variable effects of temperature on the relative mobility of the predator and the prey and the proportion of successful attacks^14^. It must be stressed that the effectiveness of the predator against mobile prey not only depends on its ability to attack and subdue a prey but also on the behaviour and defensive ability of the prey. The temperature might have differently influenced the physiology and behaviour of *B. mali* females and *T. putrescentiae* males as well as the outcomes of their interactions. When endangered, *T. putrescentiae* emits alarm pheromones and attempts to escape^81^, However, the extent to which temperature impacts pheromone production and the prey’s behavior, especially in relation to varying prey densities, remains unclear.

In our study, we found that an elevated instantaneous attack rate or increased potential for prey mortality at a given temperature did not always result in a simultaneous reduction in handling time or an increase in the maximum predation rate, making it difficult to interpret the actual impact of the predator on the *T. putrescentiae*. To address this issue, we also calculated the functional response ratio proposed by Cuthbert et al.^26^, for both the attack rate and potential of mortality. This parameter clearly showed that the impact of *B. mali* on both the eggs and males of *T. putrescentiae* intensified with rising temperatures, peaking at 35 °C.

Our findings suggest a high potential for the predatory mite *B. mali* to reduce the population of *T. putrescentiae* at higher temperatures. We determined a strong impact of temperature on predator’s efficiency as predator action accelerated under warming and increased prey consumption. The functional response type did not change with increasing temperatures; however, it changed with changing the prey stage. Although the findings provide valuable insights into the potential effectiveness of *B. mali* against *T. putrescentiae *at varying temperatures and prey stages, the scope of the study may limit its applicability to real-world scenarios. It must be stressed that under natural conditions, this predator inhabits various substrates, such as soil, litter, and decaying plant material, in which not only temperature but also humidity can affect predator and prey interaction in various ways. Moreover, substrates can vary in complexity, creating various opportunities for prey to hide and avoid predation^73^. Thus, further studies should explore the common effects of different levels of humidity and temperature as well as the role of habitat structure and prey behaviour on the functional response of *B. mali* and the stability of the predator-prey system.

## Methods

### Mite culture

The primary culture of *T. putrescentiae* reared on instant dry bakers’ yeast and wheat bran in equal parts by weight, was obtained from the mass rearing of the Department of Plant Protection, Warsaw University of Life Sciences, Warsaw, Poland^25^. Adults of *T. putrescentiae* were carefully selected and reared in glass Petri dishes measuring 90 mm in diameter, with a mixture of yeast and wheat bran in the same 50/50 ratio, to obtain 24 h eggs according to the methodology suggested by Jena et al.^25^. The colonies were kept in darkness at 26 °C and 95 ± 5% in a Sanyo Environmental Test Chamber (Panasonic MLR-350).

The stock population of *B. mali* was obtained from mass-rearing of the predator, which was maintained within wheat bran and fed on different stages of *T. putrescentiae* in the climatic room of the Department of Plant Protection at Warsaw University of Life Sciences, Warsaw, Poland. The rearing unit consisted of foam platforms, drenched in water and covered with foil within larger containers as described by Michalska et al.^41,52,53^. The cultures of *B. mali* were maintained in the Panasonic Environmental Test Chamber (MLR-352-PE), at a temperature of 23 °C, with a photoperiod of 16 h of light and 8 h of darkness and a relative humidity of 85 ± 5%.

### Functional response experiment

The experimental setup included a Plexi-glass cage with a circular hole of 8 mm diameter, a piece of filter paper affixed to the bottom of the cell, and a glass coverslip of 18 mm × 18 mm attached to the top of the cell using paraffin wax^25^. The female cohorts were prepared following the methodology described by Jena et al.^25^. The colony was fed with the mixed life stages of *T. putrescentiae* reared on yeast 24 h before choosing the female predators. Female predators were randomly chosen from the colony and exposed to varying densities, either 10, 20, 40, 60, 80, 120, or 160 eggs or 1, 2, 3, 5, 7, 10, or 15 males of *T. putrescentiae* at six different temperatures, 10 °C, 15 °C, 20 °C, 25 °C, 30 °C, and 35 °C, with a relative humidity of 85 ± 5% RH and a photoperiod of 16:8 h in an incubator (MIR-154-PE) for one day (24 h). To select the specific density of prey, pilot tests were conducted on the predation rate of *B. mali* female on *T. putrescentiae* eggs or males over one day. The separation of *T. putrescentiae *eggs from other life stages was achieved by sieving the rearing colonies through a 100 μm mesh screen and transferring eggs to the cell of the cage using a fine paintbrush^25^. The selection of *T. putrescentiae* males was done manually from the mixed population and placed in the cages with care to avoid any harm. Wet filter paper was initially placed around the hole to prevent the males from escaping, which was then replaced with a cover slip once the desired densities were achieved. After the 24 h exposure period, the predators were removed, and the consumption of eggs or males was noted, excluding any that remained. Cages where a live predator was not recovered due to loss or death were excluded from the analysis. Each egg density was replicated twenty times at each temperature, while each male density was replicated fifteen times at each temperature.

To examine the impact of temperature and prey density on the consumption of *T. putrescentiae* eggs or males by *B. mali*, we applied Generalized Linear Models (GLM) with a Poisson probability distribution. To further analyze the results, Tukey’s linear contrast was employed as a post hoc test.

The analysis of the functional response data was conducted in two phases. Initially, we focused on identifying the specific type of functional response followed by an estimation of the parameters associated with the functional response^82,72^. The functional response type was identified by applying the generalized functional response equation developed by Real^64^. The modified Holling disc equation, as proposed by Real^64^ (Eq. 1), was as follows^82^:

1 $$\:\:{\text{N}}_{\text{a}}=\:\frac{\text{a}\text{T}{\text{N}}_{0}^{(\text{q}+1)}}{1+\text{a}{\text{T}}_{\text{h}}{\text{N}}_{0}^{(\text{q}+1)}}$$

where N_a_ is the number of prey eaten, N_0_ is the initial number of prey densities provided, a is the predator’s instantaneous attack rate or searching efficiency, T_h_ is the handling time, T is the time length of the assay, and q is the scaling component that determines the shape of the curve. The functional response curve can be of different types: Type I, which is a linear relationship (q = 0 and T_h_ = 0), Type II, characterized by a hyperbolic curve (q = 0, T_h_ > 0), and Type III, displaying a sigmoid curve (q > 0, T_h_ > 0).

After identifying the appropriate shape of the functional response, the functional response parameters, i.e., instantaneous attack rate (a), handling time (T_h_), and the potential for prey mortality (α), were determined by fitting them to appropriate models. The data was then fitted to equations proposed by Roger^65^(Eq. 2), Hassell^14^ (Eq. 3), and Cabello et al.^22^ (Eq. 4), using non-linear least squares regression, as the prey that was depleted during the experiment was not replenished:

2 $$\text{Roger}\:\text{Type}\: \text{II}:{\text{N}}_{\text{a}}={\text{N}}_{0}\bigg[1-\text{exp}\bigg\{-\text{a}\text{P}\bigg(\text{T}-\frac{{\text{T}}_{\text{h}}{\text{N}}_{\text{a}}}{\text{P}}\bigg)\bigg\}\bigg]$$

3 $$\text{Hassell}\:\text{Type}\:\text{III}:{\text{N}}_{\text{a}}={\text{N}}_{0}\bigg[1-\text{exp}\bigg\{-\frac{\text{b}{\text{N}}_{0}}{1+\text{c}{\text{N}}_{0}}\bigg(\text{T}-\frac{{\text{T}}_{\text{h}}{\text{N}}_{\text{a}}}{\text{P}}\bigg)\bigg\}\bigg]$$

4 $$\text{Cabello}\:\text{et}\:\text{al.}\:\text{Type}\:\text{III}:{\text{N}}_{\text{a}}=\:{\text{N}}_{0}\:\bigg[1-\text{exp}\left\{-\frac{{\upalpha\:}{\text{N}}_{0}}{1\:+{\text{T}}_{\text{h}\:}(\text{exp}\left(-{\upalpha\:}\right)-1){\text{T}}_{\text{h}}}\left(\text{T}-\frac{{\text{T}}_{\text{h}}{\text{N}}_{\text{a}}}{\text{P}}\right)\right\}\bigg]$$

where N_a_ is the number of prey eaten, N_0_ is the initial number of prey density offered, a is the predator’s instantaneous attack rate, T_h_ is the handling time, P is the number of predators used, T is the time length of the assay, α is the potential of mortality of the predator, and b and c are the constants that relate a and N_0_ in Type III functional response as a= $\:\frac{b{N}_{0}}{1\:+c{N}_{0}}$. In our experiment, *P* = 1 and T= 1 day.

We determined the values of parameters a, T_h_, and α at all tested temperatures using a non-linear least square regression approach. The confidence intervals (± 95% CI) were calculated for these parameters, with significant differences between means indicated by non-overlapping intervals (*P* < 0.05). To calculate the Confidence Intervals (CI), we employed the permutation test method as described by Ernst^83^. Additionally, we analyzed the proportion of prey eaten by the predator at varying densities using Generalized Linear Models (GLM) with a gamma probability distribution. Further, to compare and analyze the functional response parameters, a, α, and T_h_ at six temperatures for both eggs and males as prey, the FRR was estimated by using either the attack rate (a) or potential of prey mortality (α) divided by the handling time (T_h_)^25,26^. Furthermore, the maximum predation rate^84^ was determined which is defined as the maximum number of prey that a predator consumes during a particular time frame. It was estimated by dividing the duration of the assay, T (day) by the handling time, T_h _(day). To assess whether FRRs and predation rates varied across tested temperatures, one-way Kruskal-Wallis’s rank sum tests were conducted. Post hoc comparisons were made using the Dunn test with Bonferroni corrections^25^. All statistical analyses were conducted using R version 4.3.0^85^. However, to estimate the functional response type and parameters, “FRAIR: An R Package” was applied^82^.

## Data Availability

The data used in this study are available by email request to the corresponding author MKJ (manoj_jena@sggw.edu.pl).
